# Heterotopically-placed right ventricle-to-pulmonary artery conduit does not negatively affect outcomes

**DOI:** 10.1186/s13019-023-02362-7

**Published:** 2023-09-15

**Authors:** Khunthorn Kadeetham, Piya Samankatiwat

**Affiliations:** Division of Cardiothoracic Surgery, Department of Surgery, Faculty of Medicine, Ramathibodi Hospital, Mahidol University, Bangkok, Thailand

**Keywords:** Homograft, Heterotopic position, Orthotopic position, Conduit longevity, Reintervention-free survival, Overall survival

## Abstract

**Objectives:**

Since the introduction of surgical implantation of conduit for right ventricle-to-pulmonary artery pathway reconstruction, there has been a number of studies on possible factors which might potentially affect conduit longevity, as well as patient’s reintervention-free and overall survival. Still, no definite consensual agreement could be made thus far. We aimed to compare conduit longevity, reintervention-free survival, and overall survival between patients with congenital heart diseases indicated for operations involving right ventricle-to-pulmonary artery pathway reconstruction whose conduits were placed heterotopically to those with orthotopically placed ones.

**Materials and methods:**

We retrospectively collected data from electronic medical records of Ramathibodi hospital from 1st January 2005 to 31st December 2022. Patients with congenital heart diseases whose operations involved reconstruction of right ventricle-to-pulmonary artery continuity were included. Patients whose medical record data were significantly missing were excluded. Demographic data, operative, and postoperative details were collected and reviewed.

**Results:**

There were 67 patients included in our study, with 25 receiving orthotopic and the other 42 receiving heterotopic conduit implantation. Conduit dysfunction-free, reintervention-free, and overall survival were not statistically different between both groups. There was 1 early and no late death up to the end date of our study.

**Conclusions:**

Conduits placed on a heterotopic position did not result in worse longevity, reintervention-free survival, as well as overall survival when compared to conduits placed on an orthotopic position. This suggested that the less technically demanding heterotopic conduit placement could be recommended as an operation of choice for right ventricular outflow tract reconstruction.

## Introduction

Since the introduction of operations involving creation of right ventricle-to-pulmonary artery (RV-to-PA) connection in the 1960s [1, 2], surgical techniques to restore RV-to-PA continuity have greatly evolved. As we are all aware, most conduits placed during these procedures would be on a heterotopic rather than the theoretically more hemodynamically efficient orthotopic position. There has been a number of studies [3, 4] conducted to compare outcomes between these two positions in terms of conduit longevity and patient’s reintervention-free and overall survival. However, the final results varied widely, and definite risk factors or determinants of conduit longevity, reintervention-free survival, and overall survival still could not be precisely defined.

Furthermore, not only conduit position but also conduit types and sizes could impact their durability and patient’s reintervention-free and overall survival [5–7]. However, most of the results contradicted each other, with no clear consensus made. The main limitations of these studies were the fact that there was a high heterogeneity of patients in each study, and also their limited number of patients and retrospective nature with inevitable confounders and biases.

As a result, we aimed to compare conduit longevity, reintervention-free survival, and overall survival between patients with congenital heart diseases indicated for operations involving RV-to-PA pathway reconstruction whose conduits were placed heterotopically to those with orthotopically placed ones as our primary endpoints. We also intended to identify potential risk factors for reduced conduit longevity as well as worse reintervention-free and overall survival in our patient population.

## Materials and methods

### Data collection

The study protocol and ethical issues were reviewed and approved by Human Research Ethics Committee, Faculty of Medicine, Ramathibodi Hospital, Mahidol University, Bangkok, Thailand. We retrospectively collected data from electronic medical records of Ramathibodi hospital from 1st January 2005 to 31st December 2022. The total number of operations on congenital heart diseases was 406. We included all patients with congenital heart diseases whose operations involved reconstruction of RV-to-PA continuity, whether primarily or as a correction of previous operations. Patients whose operations were not relevant and whose medical record data were significantly missing (such as no operative notes, no follow-up visits) were excluded from our study. Ultimately, 339 patients were excluded, bringing to a total of 67 patients enrolled.

Demographic data, operative (including operative time, cardiopulmonary bypass time, aortic cross-clamp time, conduit position, types, and sizes) and postoperative details (including intensive care unit stay duration, hospital stay duration, exercise capacity, and follow-up echocardiographic parameters) were collected. Time-to-conduit dysfunction and reintervention, as well as overall survival time after operation were also collected.

In our institution, we calculated body surface area (BSA) using Mosteller formula. Conduit cross-sectional area (CSA) was calculated from conduit diameter measured at the annular level. We then indexed the CSA with BSA, resulting in the term “CSA index” or “CSAi”.

### Surgical techniques

**Fig. 1:**
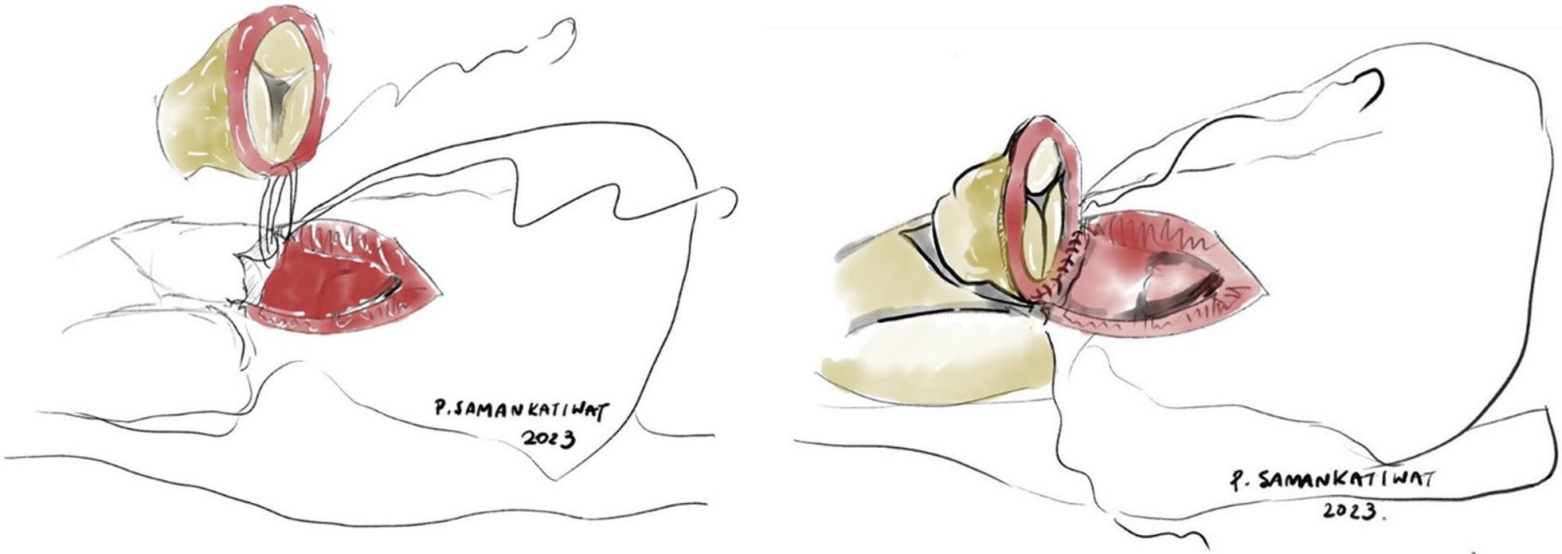
Conduit implantation on a heterotopic position. This picture illustrates the posterior one-third of the anastomosis, or the “heel”, being constructed

**Fig. 2:**
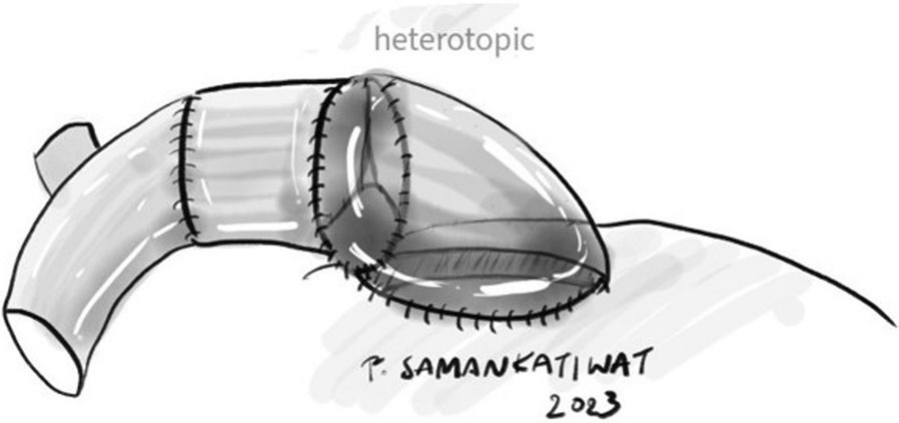
Completed heterotopic conduit implantation with the “hood” covering the anterior two-thirds of the anastomosis

For heterotopic conduit implantation, the proximal end of the conduit would be trimmed, leaving about 3 mm of tissue remaining from the valve annulus. Posterior one-third of the circumference of the proximal anastomosis, or the “heel”, would be sutured directly to the right ventriculotomy incision at epicardial level (Fig. 1). The remaining anterior two-thirds of the circumference, or the “toe”, would be reconstructed using a triangular-shaped patch made from remaining conduit material (or in some cases, a pericardial patch), thus creating the “hood”. This hood would then be sutured to the right ventriculotomy incision, also at epicardial level, completing the reconstruction (Fig. 2).

**Fig. 3:**
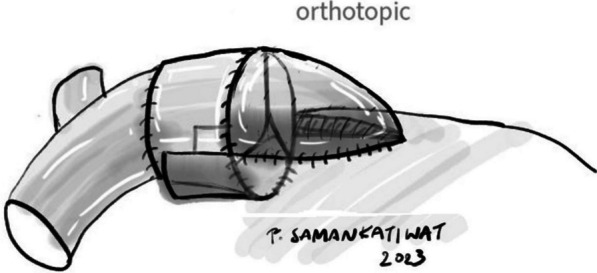
Completed orthotopic conduit implantation with its more posterior seating and an anterior patch covering the ventriculotomy incision

Orthotopic conduit implantation was mostly performed in patients with tetralogy of Fallot (TOF) who had undergone total correction with transannular patch presenting with recurrent pulmonary valve regurgitation (PVR), so-called secondary pulmonary valve replacement in our study. The transannular patch would be opened longitudinally with the regurgitant pulmonary valve subsequently excised. A new conduit was then orthotopically implanted as posteriorly as possible with its remaining exposed anterior rim covered with a patch to close the right ventriculotomy incision (Fig. 3). In cases other than post-repair TOF with recurrent PVR, or primary pulmonary valve replacement, the decision of pulmonary valve replacement would be made in the first place if the pulmonary annulus was extremely small (z-score < − 3). The remaining  steps of operation were generally the same. Right ventricular outflow tract (RVOT) enlargement would then be necessary to accommodate a new conduit. All xenografts in our study were orthotopically implanted.

For patients with concomitant pulmonary artery stenosis, we routinely performed pulmonary arterioplasty using either glutaraldehyde-fixed autologous or bovine pericardium as an enlargement patch. Techniques for other concomitant procedures would not be described in details in this study.

**Fig. 4:**
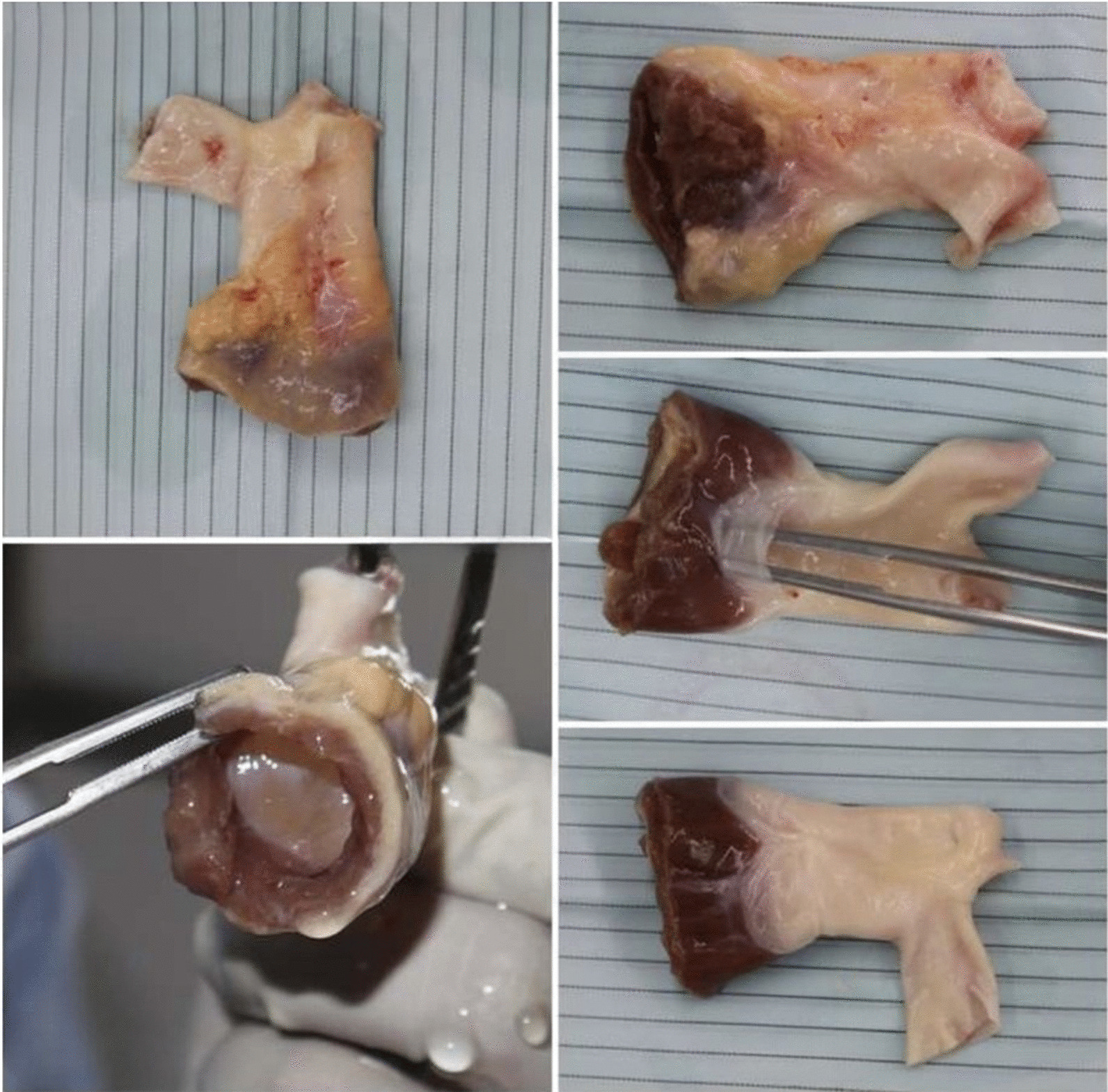
An example of a pulmonary homograft supplied by The Thai Red Cross Organ Donation Center

Conduit type and size were mainly selected by the operating surgeon based on the individual’s preferences and conduit availability. Homografts which were deemed too oversized (more than + 2SD of the BSA) but with no smaller ones available would be bicuspidized in order to reduce their diameter to about two-thirds the original. We analyzed the final diameter after bicuspidization as the actual conduit diameter in our study (Fig. 4).

### Follow-up

Postoperative echocardiographic evaluation was performed at 6 months to 1 year postoperatively. Subsequent evaluation would be performed half-yearly or yearly thereafter. We defined conduit dysfunction as having either moderate-to-severe degree of conduit stenosis (peak systolic pressure gradient across conduit > 40 mmHg) or moderate-to-severe degree of conduit regurgitation from echocardiographic parameters [8]. Conduit calcification alone was not considered as conduit dysfunction. Reintervention was considered on a case-by-case basis in our hospital. Both surgical and percutaneous pulmonary valve replacement were considered as reintervention. Exercise capacity was evaluated during out-patient department visits by the attending physicians. Patients whose last follow-up visit date was beyond the end date of study were considered to be alive while others whose last visit was within the study period were verified to be alive by contacting them or their relatives with telephone calls.

### Statistical analysis

Patient characteristics with continuous variables were compared using Student’s t-test while categorical variables were compared with chi-square or Fisher’s exact tests. P-value of less than 0.05 was considered statistically significant. Potential risk factors were analyzed by univariate and multivariate methods using Cox regression model. Independent risk factors were expressed as hazard ratios (HR) with 95% CIs. Survival analyses were performed using Kaplan–Meier method and evaluated with log-rank test. The statistical software used was Stata version 14.1.

## Results

### Patient characteristics (Table 1)

There were 67 patients included in our study, with 25 in orthotopic and 42 in heterotopic position group. Patients who underwent orthotopic conduit implantation were significantly older (median 18 vs. 4 years old, P < 0.001) and had generally bigger body sizes compared to those who underwent heterotopic conduit implantation (P < 0.001 for all variables). Also, there were significantly more patients diagnosed with TOF (who subsequently underwent total correction) in orthotopic position group (16 vs. 4, P < 0.001). Systemic-to-pulmonary shunt procedures were performed significantly more in heterotopic position group (23 vs. 2, P < 0.001) as the major diagnosis was pulmonary atresia and ventricular septal defect (PA/VSD) (52.38%). Co-morbidities were generally similar between both groups. All patients diagnosed with persistent truncus arteriosus (TA) underwent heterotopic conduit implantation as compulsorily required.

**Table 1:** Patient characteristics

Variables	Orthotopic (N = 25)	Heterotopic (N = 42)	P-value
Age at operation: Median (P25,P75)	18 (14, 27)	4 (1, 6)	< 0.001
Age ≤ 15 years	9 (36.00)	39 (92.86)	< 0.001
Age > 15 years	16 (64.00)	3 (7.14)	
Sex: N (%)			
Male	12 (48.00)	20 (47.62)	0.976
Female	13 (52.00)	22 (52.38)	
Height (cm): Median (P25,P75)	156.5 (150.5, 161.5)	103.0 (82.0, 126.0)	< 0.001
Weight (kg): Median (P25,P75)	47.0 (35, 53)	13.6 (9.5, 16)	< 0.001
BSA (m^2^): Median (P25,P75)	1.42 (1.3, 1.62)	0.56 (0.45, 0.72)	< 0.001
Congenital heart disease diagnosis			
TOF	16 (64.00)	4 (9.52)	< 0.001
DORV/PS	8 (32.00)	13 (30.95)	
PA/VSD	1 (4.00)	22 (52.38)	
TA	0 (0.00)	3 (7.14)	
Previous surgeries: N (%)			
Systemic-to-pulmonary shunt	2 (8.00)	23 (54.76)	< 0.001
Total correction of TOF	18 (72.00)	1 (2.38)	
Unifocalization	0 (0.00)	2 (4.76)	
MAPCAs ligation	0 (0.00)	2 (4.76)	
Co-morbidities: N (%)			
DiGeorge syndrome	1 (4.00)	2 (4.76)	0.999
Down syndrome	1 (4.00)	0 (0.00)	
LPA stenosis	0 (0.00)	1 (2.38)	
Atrial fibrillation	1 (4.00)	0 (0.00)	
PAPVC	0 (0.00)	1 (2.38)	
Severe rheumatic aortic regurgitation	0 (0.00)	1 (2.38)	

BSA, body surface area; TOF, tetralogy of Fallot; DORV/PS, double outlet right ventricle and pulmonary stenosis; PA/VSD, pulmonary atresia and ventricular septal defect; TA, truncus arteriosus; MAPCAs, major aortopulmonary collateral arteries; LPA, left pulmonary artery; PAPVC, partial anomalous pulmonary venous connection

### Operative details (Table 2)

Heterotopic conduit implantation was associated with longer operative time compared to orthotopic conduit implantation (median 350 vs. 315 min, P < 0.05). Similarly, heterotopic conduit implantation was also associated with longer cardiopulmonary bypass time (median 167 vs. 114 min, P < 0.001) and aortic cross-clamp time (median 120.5 vs. 0 min, P < 0.001) as all orthotopic conduit implantation procedures were performed on a beating heart basis. The median conduit size and cross-sectional area were significantly larger in orthotopic position group (median 23 vs. 19, P < 0.001 and median 415.27 vs. 283.39 mm^2^, P < 0.001, respectively) than in heterotopic position group. In terms of conduit type, the use of pulmonary homografts was similar between the two groups. There was no significant difference between the two groups in terms of conduit bicuspidization (total of 4 cases, all in heterotopic position group) or the concomitant procedures performed. The patient who had undergone concomitant aortic valve replacement had rheumatic severe aortic regurgitation along with double outlet right ventricle and severe pulmonary stenosis with no aortic root involvement, therefore, the operation was Rastelli operation with concomitant bioprosthetic aortic valve replacement.

**Table 2:** Operative details

Variables	Orthotopic (N = 25)	Heterotopic (N = 42)	P-value
Operative time (mins): Median (P25,P75)	315 (260, 360)	350 (315, 440)	< 0.05
Cardiopulmonary bypass time (mins): Median (P25,P75)	114 (82, 139)	167 (145,207)	< 0.001
Aortic cross-clamp time (mins): Median (P25,P75)	0 (0, 104)	120.5 (103, 136)	< 0.001
Conduit type: N (%)			
Pulmonary homograft	16 (64.00)	22 (52.38)	< 0.05
Aortic homograft	0 (0.00)	11 (26.19)	
Contegra (Medtronic, Inc., Minneapolis, MN, USA)	5 (20.00)	8 (19.05)	
Xenograft	4 (16.00)	1 (2.38)	
Conduit size (mm): Median (P25,P75)	23 (22, 26)	19 (17, 21)	< 0.001
Conduit cross-sectional area (mm^2^): Median (P25,P75)	415.27 (379.94, 530.66)	283.39 (226.87, 346.19)	< 0.001
Conduit bicuspidization: N (%)			
Yes	0 (0.00)	4 (9.52)	0.112
No	25 (100.00)	38 (90.48)	
Concomitant procedures: N (%)			
Pulmonary arterioplasty	6 (24.00)	14 (33.33)	0.227
Pulmonary artery dilation	1 (4.00)	0 (0.00)	
Tricuspid valve repair	1 (4.00)	1 (2.38)	
Truncal valve repair	0 (0.00)	3 (7.14)	
Aortic valve replacement	1 (4.00)	1 (2.38)	
RVOT reconstruction	1 (4.00)	0 (0.00)	
Residual VSD closure	1 (4.00)	0 (0.00)	
PAPVC repair	0 (0.00)	1 (2.38)	

RVOT, right ventricular outflow tract; VSD, ventricular septal defect; PAPVC, partial anomalous pulmonary venous connection

### Postoperative details (Table 3)

There was no significant difference in hospital stay between the orthotopic and the heterotopic position group (median 7 vs. 10 days, P = 0.086). However, the heterotopic position group had a significantly longer intensive care unit (ICU) stay compared to the orthotopic position group (median 5 vs. 3 days, P < 0.05). There was also no significant difference in exercise capacity assessed at 6 months postoperatively between the two groups with most patients from both groups having improved postoperative exercise capacity (76% in orthotopic and 85.71% in heterotopic position group). Regarding echocardiographic parameters, the heterotopic position group had a significantly larger left ventricular end-diastolic volume index (LVEDVI) compared to the orthotopic position group (median 63.69 vs. 51.90 ml/m^2^, P < 0.05). However, there were no significant differences in left ventricular end-systolic volume index (LVESVI), left ventricular ejection fraction (LVEF), fractional shortening (FS), and also tricuspid annular plane systolic excursion (TAPSE) between both groups. Postoperative peak systolic pressure gradient (PSPG) across conduit was also similar between both groups. There was a total of 26 conduit dysfunctions (12 in orthotopic and 14 in heterotopic group). Of the 12 patients with conduit dysfunction in the orthotopic group, 11 had moderate-to-severe conduit regurgitation while only 1 had moderate-to-severe conduit stenosis. On the other hand, of the 14 patients with conduit dysfunction in the heterotopic group, 10 had moderate-to-severe conduit regurgitation while the other 4 had moderate-to-severe conduit stenosis.

**Table 3:** Postoperative details

Variables	Orthotopic (N = 25)	Heterotopic (N = 42)	P-value
Hospital stay (days): Median (P25,P75)	7 (6, 13)	10 (7, 14)	0.086
ICU stay (days): Median (P25, P75)	3 (2, 5)	5 (4, 6)	< 0.05
Exercise capacity at 6 months postoperatively: N (%)			
Improved	19 (76.00)	36 (85.71)	0.316
Same	6 (24.00)	6 (14.29)	
Worsened	0 (0.00)	0 (0.00)	
Echocardiographic parameters at 6 months–1 year postoperatively			
LV function: Median (P25,P75)			
LVEF (%)	63.65 (55, 72)	67.85 (63.25, 75.60)	0.087
FS (%)	33.85 (30.3, 41.6)	37.20 (35, 44)	0.129
LV volume: Median (P25, P75)			
LVEDVI (ml/m2)	51.90 (37.27, 66.79)	63.69 (56.82, 85.00)	< 0.05
LVESVI (ml/m2)	17.47 (10.29, 26.97)	23.48 (14.88, 31.89)	0.418
RV function: Median (P25, P75)			
TAPSE (cm)	1.69 (1.50, 2.01)	1.52 (1.17, 1.70)	0.079
PSPG across conduit (mmHg): Median (P25, P75)	19.90 (14, 25)	20.00 (8.0, 32.0)	0.640
Conduit dysfunction: N (%)			
Conduit stenosis	1 (4.00)	4 (9.52)	0.302
Conduit regurgitation	11 (44.00)	10 (23.81)	
No conduit dysfunction	13 (52.00)	23 (54.76)	

ICU, intensive care unit; LV, left ventricle; LVEF, left ventricular ejection fraction; FS, fractional shortening; LVEDVI, left ventricular end-diastolic volume index; LVESVI, left ventricular end-systolic volume index; RV, right ventricle; TAPSE, tricuspid annular plane systolic excursion; PSPG, peak systolic pressure gradient

### Comparison of conduit size, cross-sectional area, and cross-sectional area index between patients with and without conduit dysfunction (Table 4)

The results showed that patients with conduit dysfunction had significantly larger conduit size and cross-sectional area than those without conduit dysfunction (mean 22 vs. 19 mm, P < 0.05 and mean 379.94 vs. 283.39 mm^2^, P < 0.05, respectively). However, the conduit cross-sectional area index did not differ significantly between the two groups (mean 371.69 vs. 404.84 mm^2^/m^2^, P = 0.987).

**Table 4:** Comparison of conduit size, cross-sectional area, and cross-sectional area index between patients with and without conduit dysfunction

Variables	Conduit dysfunction (stenosis/regurgitation) (N = 26)	No conduit dysfunction (N = 41)	P-value
Conduit size (mm): Median (P25, P75)	22 (18, 25)	19 (16.5, 22)	< 0.05
Conduit cross-sectional area (mm^2^): Median (P25, P75)	379.94 (254.34, 490.63)	283.39 (213.72, 379.94)	< 0.05
Conduit cross-sectional area index (mm^2^/m^2^): Median (P25, P75)	371.69 (336.63, 479.89)	404.84 (312.23,515.25)	0.987

### Risk factors associated with conduit dysfunction, reintervention, and overall survival (Tables 5, 6, 7)

The results demonstrated that age at operation of less than 15 years and conduit size of less than 20 mm were not associated with higher risk of conduit dysfunction, reintervention, and worse overall survival. Orthotopic conduit position was not statistically significantly associated with higher risk of conduit dysfunction (HR 1.459, P = 0.368), reintervention (HR 1.755, P = 0.178), and worse overall survival (HR 1.50, P = 0.322). Diagnosis of double outlet right ventricle and pulmonary stenosis (DORV/PS) was associated with higher rate of conduit dysfunction (HR 7.653, P < 0.001) and reintervention (HR 9.38, P < 0.001), but not worse overall survival according to univariate analysis. Different types of previous surgeries were not significantly associated with better or worse outcomes.

**Table 5:** Univariate and multivariate analyses demonstrating factors associated with conduit dysfunction

Variables	Univariate analysis			Multivariate analysis		
	HR	95% CI	P-value	HR	95% CI	P-value
Age at operation						
Age ≤ 15 years	0.586	0.25 to 1.38	0.223	0.190	0.04 to 0.96	< 0.05
Age > 15 years						
Conduit size						
≤ 20 mm	1.01	0.44 to 2.30	0.989	–	–	–
> 20 mm						
Conduit type						
Pulmonary homograft						
Aortic homograft	0.814	0.24 to 2.78	0.742	0.613	0.09 to 4.13	0.615
Contegra (Medtronic Inc., Minneapolis, MN, USA)	2.871	0.87 to 9.46	0.083	2.859	0.38 to 21.66	0.309
Xenograft	0.123	0.02 to 0.98	0.047	0.123	0.01 to 1.12	0.063
Conduit position						
Orthotopic	1.459	0.64 to 3.32	0.368	–	–	–
Heterotopic						
BSA (m^2^)	0.652	0.28 to 1.54	0.331	–	–	–
Congenital heart disease diagnosis						
TOF						
DORV/PS	7.653	2.55 to 23.01	< 0.001	4.404	1.16 to 16.77	< 0.05
PA/VSD	1.846	0.68 to 5.02	0.230	1.01	0.10 to 10.74	0.994
TA	–	–	–	–	–	–
Previous surgeries						
Systemic-to-pulmonary shunt				–	–	–
Total correction of TOF	0.611	0.24 to 1.59	0.312	1.680	0.13 to 20.98	0.687
Unifocalization	–	–	–	–	–	–
MAPCAs ligation	1.692	0.20 to 14.39	0.630	0.973	0.08 to 12.22	0.983

TOF, tetralogy of Fallot; DORV/PS, double outlet right ventricle and pulmonary stenosis; PA/VSD, pulmonary atresia and ventricular septal defect; TA, truncus arteriosus; MAPCAs, major aortopulmonary collateral arteries

**Table 6:** Univariate and multivariate analyses demonstrating factors associated with reintervention

Variables	Univariate analysis			Multivariate analysis		
	HR	95% CI	P-value	HR	95% CI	P-value
Age at operation						
Age ≤ 15 years	0.435	0.18 to 1.03	0.059	0.904	0.12 to 6.75	0.921
Age > 15 years						
Conduit size						
≤ 20 mm	0.667	0.29 to 1.56	0.348	–	–	–
> 20 mm						
Conduit type						
Pulmonary homograft						
Aortic homograft	1.976	0.67 to 5.86	0.219	1.834	0.34 to 9.80	0.478
Contegra (Medtronic Inc., Minneapolis, MN, USA)	2.486	0.78 to 7.95	0.125	1.821	0.37 to 9.06	0.464
Xenograft	0.143	0.02 to 1.10	0.062	0.166	0.02 to 1.36	0.094
Conduit position						
Orthotopic	1.755	0.78 to 3.98	0.178	0.652	0.14 to 3.14	0.594
Heterotopic						
BSA (m^2^)	0.297	0.12 to 0.77	< 0.05	0.846	0.15 to 4.76	0.850
Congenital heart disease diagnosis						
TOF						
DORV/PS	9.38	2.70 to 32.60	< 0.001	9.22	1.22 to 69.99	< 0.05
PA/VSD	2.95	1.04 to 8.35	< 0.05	12.12	0.53 to 27.86	0.118
TA	–	–	–			
Previous surgeries						
Systemic-to-pulmonary shunt				–	–	–
Total correction of TOF	0.414	0.16 to 1.11	0.079	1.649	0.11 to 24.77	0.718
Unifocalization	–	–	–	–	–	–
MAPCAs ligation	1.08	0.13 to 8.82	0.943	0.829	0.07 to 10.49	0.885

TOF, tetralogy of Fallot; DORV/PS, double outlet right ventricle and pulmonary stenosis; PA/VSD, pulmonary atresia and ventricular septal defect; TA, truncus arteriosus; MAPCAs, major aortopulmonary collateral arteries

**Table 7:** Univariate and multivariate analyses demonstrating factors associated with overall survival

Variables	Univariate analysis			Multivariate analysis		
	HR	95% CI	P-value	HR	95% CI	P-value
Age at operation						
Age ≤ 15 years	0.483	0.21 to 1.12	0.091	0.494	0.10 to 2.40	0.382
Age > 15 years						
Conduit size						
≤ 20 mm	0.422	0.17 to 1.03	0.058	1.708	0.36 to 8.04	0.498
> 20 mm						
Conduit type						
Pulmonary homograft						
Aortic homograft	0.814	0.24 to 2.78	0.742	0.611	0.36 to 1.03	0.064
Contegra (Medtronic Inc., Minneapolis, MN, USA)	2.87	0.87 to 9.46	0.083			
Xenograft	0.123	0.02 to 0.98	0.047			
Conduit position						
Orthotopic	1.50	0.67 to 3.34	0.322			
Heterotopic						
BSA (m^2^)	0.124	0.04 to 0.40	< 0.05	0.082	0.01 to 0.57	< 0.05
Congenital heart disease diagnosis						
TOF	1.399	0.92 to 2.14	0.121	0.909	0.45 to 1.84	0.791
DORV/PS						
PA/VSD						
TA						
Previous surgeries						
Systemic-to-pulmonary shunt	0.889	0.41 to 1.95	0.769	–	–	–
Total correction of TOF						
Unifocalization						
MAPCAs ligation						

TOF, tetralogy of Fallot; DORV/PS, double outlet right ventricle and pulmonary stenosis; PA/VSD, pulmonary atresia and ventricular septal defect; TA, truncus arteriosus; MAPCAs, major aortopulmonary collateral arteries

### Conduit dysfunction-free, reintervention-free, and overall survival (Fig. 5)

According to the Kaplan–Meier survival curves shown below, conduit dysfunction-free, reintervention-free, and overall survival were estimated to be statistically the same between patients who underwent heterotopic and orthotopic conduit implantation (P = 0.364, P = 0.172, and P = 0.319, respectively). The median time for conduit dysfunction-free survival was 123 months in orthotopic and 85 months in heterotopic group. For reintervention-free survival, it was 126 months in orthotopic and 96 months in heterotopic group. And lastly for overall survival, it was 144 months in orthotopic and 96 months in heterotopic group. Considering all patients in our cohort from both groups, the median time for conduit dysfunction-free survival, reintervention-free survival, and overall survival was 126, 191, and 78 months, respectively. The odd results were due to the increased heterogeneity of time when both groups were combined. There was 1 early (in-hospital) death due to postoperative pulmonary hypertensive crisis with progressive right ventricular failure and no late death.

**Fig. 5:**
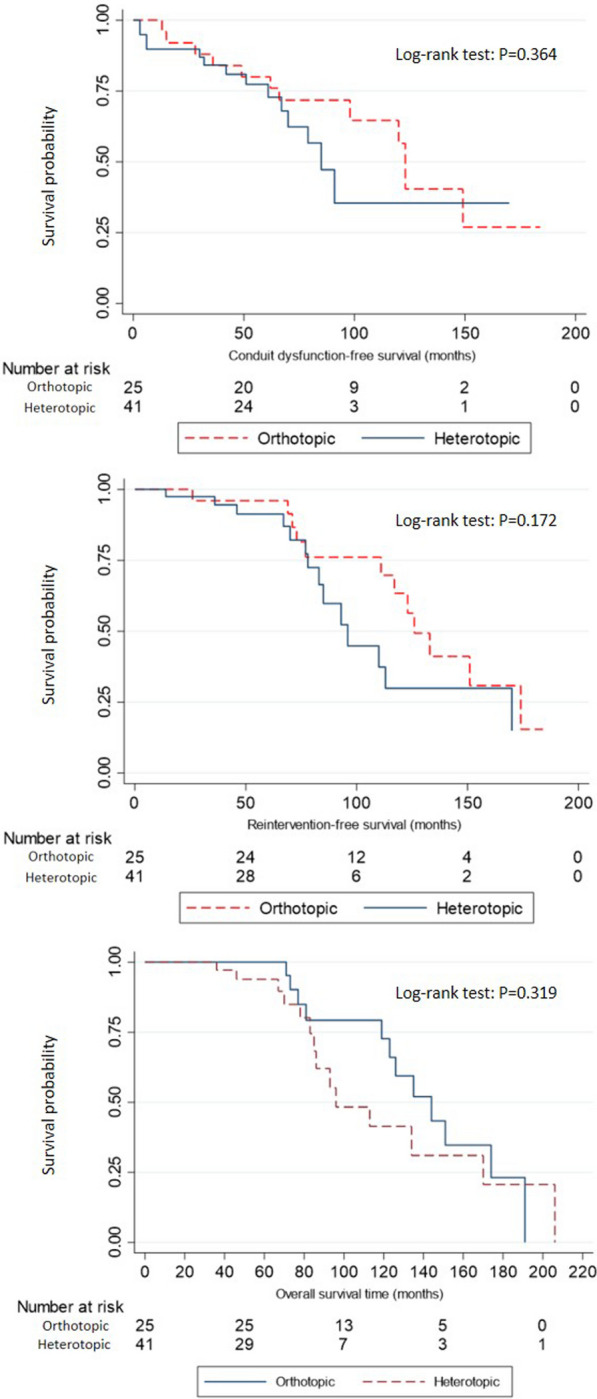
Kaplan–Meier survival analyses demonstrating conduit dysfunction-free, reintervention-free, and overall survival time between orthotopic and heterotopic conduit implantation

## Discussion

The history of surgical creation of right ventricle to pulmonary artery continuity could be traced back to the 1960s when Ross et al. [1] and Rastelli et al. [2] pioneered operations involving implantation of a conduit to connect the right ventricle to the main pulmonary artery in patients diagnosed with PA/VSD. Conduits placed for such purposes were usually on a heterotopic position, rather than the theoretically more hemodynamically efficient orthotopic position like in a normal heart. This led to suggestion that a heterotopically-placed conduit position might negatively affect its function and longevity, which could possibly result in the need for reintervention and ultimately to decreased patient’s overall survival.

Techniques for surgical placement of a conduit on an orthotopic position are more demanding, as enlargement of the RVOT to accommodate the new, larger conduit would be necessary. Sometimes, the size of the newly-enlarged RVOT would still not be compatible to the conduits available, forcing surgeons to implant smaller-than-ideal conduits. Therefore, the technically simpler and more straightforward heterotopic conduit placement seems to be an interesting and more feasible option. However, there has only been a handful of studies directly conducted on this topic regarding the heterotopic conduit position on its longevity and possible negative effects on the patient’s need for reintervention and overall survival. One study [3] compared conduit longevity and overall survival of patients at 15 years after placement of conduits on an orthotopic to a heterotopic position. There were no significant differences in both groups. On the other hand, another study [4] yielded an opposite result, with patients receiving conduits placed heterotopically having worse reintervention-free survival than those receiving conduits placed orthotopically. The reasons behind these conflicting results had been debated by both authors, stating that in both studies, patients who received conduits placed orthotopically were older and had significantly bigger body sizes, resulting in the possibility to receive larger or “oversized” conduits. Theoretically, an oversized conduit might mitigate the patient’s “outgrowth” process, which would render the conduit relatively too small for the eventual body size.

Conduit type and size, as well as patient’s age at operation, have been thought to have significant effects on conduit longevity [5–10]. Our results found that there were no associations between younger ages at operation (age less than 15 years) or smaller conduit sizes (diameter less than 20 mm) and more conduit dysfunction or worse reintervention-free and overall survival. The reason was that in our study, every patient would have already received an oversized conduit according to BSA (not just numerically large). In our institution, we routinely implanted oversized conduits, albeit no more than + 2SD the ideal size for the corresponding BSA according to the nomogram [11]. We found that oversized conduits resulted in acceptable reintervention-free and overall survival. We could suggest that placing oversized conduits might in fact be beneficial as we believed that patient “outgrowing” the conduit was a significant risk factor for reintervention. Importantly, large and oversized conduits could be more easily placed on a heterotopic position compared to an orthotopic position, which would otherwise require extensive RVOT enlargement. Limitation regarding smaller homograft sizes availability resulted in the necessity of conduit bicuspidization in some cases. We also found bicuspidization to be a satisfactory solution as none of our patients who received bicuspidized homograft conduits ended up with conduit dysfunction or reintervention.

However, some believed that oversized conduits were associated with higher rate of conduit kinking and narrowing from sternal compression. As a result, these conduits were more prone to turbulent flow, which might increase their wall shear stress and cause earlier deterioration [12–14]. Coronary compression and pulmonary arteries distortion [15] from conduit compression might also occur. This could ultimately result in earlier conduit stenosis. Even that said, we still believed that if conduits were not too oversized and placed on a proper position away from the sternal table, conduit kinking or compression by the sternum could be avoided.

A few studies [16, 17] evaluated patients who previously underwent TOF repair during childhood with pulmonary valve regurgitation (PVR). Patients had their regurgitant pulmonary valves replaced either surgically or percutaneously. Their results suggested that pulmonary valve replacement in patients with post-TOF repair PVR resulted in reduced ventricular dimensions and improved ventricular function. Our study also included a significant number of patients with post-TOF repair PVR who underwent secondary pulmonary valve replacement by an orthotopically-placed conduit. In our study, according to the acquired results, we compared ventricular function and exercise capacity between patients who had conduits placed orthotopically and heterotopically. Heterotopically placed conduits, which resulted in more turbulent blood flow, might theoretically adversely affect long-term ventricular function. However, our results proved the contrary, with both groups yielding similar results. As there were more patients with secondary pulmonary valve replacement in the orthotopic position group than the heterotopic position group, we suspected that the possibly beneficial effect in terms of efficiency of  orthotopic conduit position might be nullified when faced with the fact that this group of patients might already have had worse cardiac function. These parameters might not improve as well as in patients who had normal or near-normal ventricular function before surgery.

Pulmonary hypertension could also affect conduit function. As in our study, the majority of patients receiving RV-to-PA conduit reconstruction were diagnosed with either TOF, PA/VSD, or DORV/PS. These diseases on their own generally resulted in restricted pulmonary blood flow, which would not result in pulmonary vascular obstructive disease (PVOD). Therefore, they might not have negative effects on conduit function and longevity. However, with the rarer diagnosis of TA, which resulted in pulmonary overflow, the results might prove otherwise. Our results did not demonstrate TA to be a potential risk factor for conduit dysfunction, reintervention-free survival, and overall survival. We believed that the reason behind this was that patients with TA generally underwent surgical conduit placement much earlier in life, in which PVOD had not yet developed [18, 19]. Also, we had very limited number of TA patients in our cohort, which might not be enough to be statistically significant. We also believed that concomitant pulmonary arterioplasty would result in reduction of postoperative pulmonary artery pressure. This should theoretically reduce conduit afterload and therefore resulted in longer conduit dysfunction-free survival.

A few studies [20–23] compared pulmonary and aortic homograft in terms of reintervention-free and overall survival. Their results suggested that pulmonary homograft use was associated with better reintervention-free and overall survival compared to aortic homograft. They suggested that because aortic homografts had more elastic tissue and tissue calcium content, this would result in more conduit calcification and stenosis. Even though our results did not show pulmonary homograft to be superior in terms of conduit dysfunction-free, reintervention-free, and overall survival, we still preferred pulmonary homograft to be the conduit of choice at our institution. One study mentioned that immune process had a more prominent role in causing conduit dysfunction in children than in adults. However, from our results, we found that younger age at operation did not adversely affect conduit longevity, reintervention-free survival, and overall survival. We could suggest that immune process was not a risk factor in determining either conduit longevity or reintervention-free and overall survival.

Decellularized homografts, on the basis that they lacked living cells, would result in less immune reactions and possibly longer conduit durability [24, 25]. Other studies concentrated on the preservation processes of homografts, including immersing in antibiotic solution [26], the now-obsolete irradiation treatment, and the most commonly used cryopreservation technique. In our hospital, we routinely used cryopreserved homografts supplied by Thai Red Cross Organ Donation Center (Fig. 4). Homografts would be harvested, soaked in 200 ml of Roswell Park Memorial Institute (RPMI) 1640 solution, and preserved in 90 ml of Medium 199 solution combined with 10 ml of 10% dimethyl sulfoxide (DMSO) solution (Thermo Fisher Scientific, Waltham, Massachusetts, USA). They would then be cryopreserved and stored in liquid nitrogen at -196 degrees Celsius, with a shelf life of 5 years. Even with different processing and preservation techniques as compared to others’, we could still obtain reasonably good results from our patients.

Cryopreservation theoretically would result in elimination of living cells in conduits, leaving only the “scaffolding” remaining [27]. From this aspect, it was implied that immune reaction could not have been the cause of conduit deterioration, as there were no living cells for the immune system to attack. This supported our idea that not immune, but outgrowth process, was the main determinant of conduit longevity. However, one study [28] found that most of their patients whose conduits failed were from conduit constriction and shrinking, not from outgrowth process, suggesting that immune process was the main risk factor. Although their results were convincing, we believed that conduit constriction and shrinking could have also been from other factors other than immune process.

Surgical technique-wise for heterotopic conduit implantation, we routinely sutured proximal conduit anastomosis to the right ventricular (RV) epicardium [29], not buried deep to the infundibular septum. Even with this technique, which could possibly place the conduit at higher risk of being compressed by the sternum because of its more anterior position, we found that our heterotopically-placed conduits were not associated with worse durability, reintervention-free survival, or overall survival as compared to the orthotopically-placed ones. We could state from our results that the simpler RV epicardial proximal anastomotic suturing technique could be adapted as the outcomes were satisfactory.

We calculated conduit CSA [30] indexed to BSA, creating “CSA index” (CSAi), in our study. We believed that this term would be the most accurate in comparing patients with different conduit sizes as they would have different BSAs. By indexing CSA with BSA, we could obtain a more standardized term corresponding to the individual patient’s BSA for direct comparison rather than conduit diameter or CSA alone. Our aim was to determine if patients whose conduits failed had significantly higher CSAi when compared to patients whose conduits did not. Our results demonstrated that patients in the failure group did not have conduits with significantly higher CSAi implanted compared to those in the non-failure group. This would strongly suggest that implantation of oversized conduits would not result in their reduced longevity or decreased reintervention-free and overall survival, as supported by other findings in our study.

## Limitations

Bias could not be fully eliminated as this was a retrospective trial. Also, we did not have a regularly-scheduled echocardiographic follow-up appointment protocol in our hospital. This was mainly due to the availability of the echocardiography laboratory and also the attending pediatric cardiologists. Another limitation regarding echocardiography was the recorded parameters in which some were either not mentioned or lost, resulting in incomplete postoperative echocardiographic parameters for our final analysis.

Secondly, as mentioned earlier, choice of operation and conduit type were mainly made based on the operating surgeon’s preferences, resulting in possible biases. Also, there were no clearly defined indications for reintervention in patients with conduit dysfunction at our institution. This could probably result in either over- or under-reintervention for our patients.

Lastly, as with many other studies, we had limited number of patients included in our study. With diagnoses of TOF (who needed RV-to-PA reconstruction), DORV/PS, and TA being relatively rare compared to other kinds of congenital heart diseases or adult cardiac diseases, larger patient population would be too difficult to come by. Also, randomized-controlled trials on this topic would be too time-costly or even nearly impossible to realistically conduct.

## Conclusions

As for creation of RV-to-PA continuity, conduits placed on a heterotopic position did not result in worse longevity, reintervention-free survival, as well as overall survival when compared to conduits placed on an orthotopic position. The less technically complicated heterotopic conduit placement could be strongly recommended as an operation of choice for RVOT reconstruction according to our results. Also, choosing a moderately oversized conduit would be advisable to mitigate the degree of patient outgrowing the conduit, which we believed to be an important risk factor for conduit dysfunction. Lastly, we encouraged the use of CSAi as a useful new term to accurately and reliably compare between different conduit sizes.

## Data Availability

Not applicable.
